# Efficient Copper Removal from an Aqueous Anvironment using a Novel and Hybrid Nanoadsorbent Based on Derived-Polyethyleneimine Linked to Silica Magnetic Nanocomposites

**DOI:** 10.3390/nano9020209

**Published:** 2019-02-06

**Authors:** Olivija Plohl, Matjaž Finšgar, Sašo Gyergyek, Urban Ajdnik, Irena Ban, Lidija Fras Zemljič

**Affiliations:** 1 Laboratory for Characterization and Processing of Polymers, University of Maribor, Faculty of Mechanical Engineering, Smetanova 17, 2000 Maribor, Slovenia; urban.ajdnik@um.si (U.A.); lidija.fras@um.si (L.F.Z.); 2 Faculty of Chemistry and Chemical Engineering, University of Maribor, Smetanova 17, 2000 Maribor, Slovenia; matjaz.finsgar@um.si (M.F.); saso.gyergyek@ijs.si (S.G.); irena.ban@um.si (I.B.); 3 Department for Materials` Synthesis, Jožef Stefan Institute, Jamova 39, 1000 Ljubljana, Slovenia

**Keywords:** environmental nanotechnology, nanoparticle characterization, surface analysis, magnetic polymer nanosorbents, heavy metal reduction, hybrid nanocomposites

## Abstract

Due to the extreme rise of sludge pollution with heavy metals (e.g. copper), the options for its disposal or treatment are decreasing. On the contrary, properly heavy metal-cleaned sludge can be used as an alternative sustainable energy and agriculture source. The aim of this study was to develop a novel nanoadsorbent, based on irreversibly linked amino-rich polymer onto previously silica-coated magnetic nanoparticles (MNPs) that can be applied efficiently for metal removal. MNPs were coated uniformly by 3 nm thick silica layer (core-shell structure), and were additionally modified with systematic covalent attachment of derived branched polyethyleneimine (bPEI). The formed structure of synthesized MNPs composite was confirmed with several analytical techniques. Importantly, nanoadsorbents exhibit high density of chelating amino groups and large magnetic force for easier separation. The importance of introduced bPEI, effect of pH, initial heavy metal concentration onto copper uptake efficiency and, further, nanoadsorbent regeneration, were studied and explained in detail. The adsorption isotherm was well fitted with Langmuir model, and the maximum adsorption capacity was shown to be 143 mg·g^−1^ for Cu^2+^. The reusability and superior properties of silica-coated MNPs functionalized with derived-bPEI for copper adsorption underlie its potential for the removal application from heavy metals contaminated sludge

## 1. Introduction

During recent decades, the upgrade of municipal wastewater treatment plants has led to increased sludge production. Consequently, sludge quantities continue to increase exponentially, whereas disposal options are decreasing due to their contamination with inorganic pollutants, such as Heavy Metals (HMs), by obeying strict national legislation. For instance, in the EU, it is estimated that sludge production will reach approximately 13 million tons of dried matter by 2020. On the contrary, moderately HM-cleaned sewage sludge can be used beneficially on lands as a fertilizer or as a soil conditioner, which can be advantageous by providing an economical, renewable disposal alternative, together with the additional nutritious supplement for plants [1,2,3,4,5]. Although the contamination with specific HMs is dependent on the location and type of the produced sludge, the major HM contaminant in significantly higher amount, as the legislation allows, is copper [1]. Nowadays, copper is used widely in various manufacturing industries (e.g., Electroplating), which caused its huge accumulation in the sewage sludge [6]. Besides, copper can bio-accumulate in plants, and can be deposited in the human body via the food chain. Despite the known necessity of the latter as an indispensable element for plants and humans, an excessive amount of copper can result in severe health problems [7]. Following this issue, there is an urgent need to remove the excess copper from sewage sludge to provide recycling (zero waste concept) and economical disposal of sludge with high nutrients values (i.e., phosphorus, nitrogen, organic matter) for agricultural land applications. 

To date, several conventional methods for HMs` removal from contaminated wastewater have been introduced, such as chemical precipitation, coagulation, ultrafiltration, and adsorption [8,9,10]. Among them, a lot of attention has been paid to the adsorption process, as it allows higher efficiency, lower operational costs accompanied with lower energy use, process reversibility and flexibility in adsorbent design [11,12]. Most of the newly established adsorbents were based on active carbon, graphene oxide, mesoporous silica, or zeolites [11]. However, their major drawback is their complicated removal after the adsorption process (filtration, centrifugation), correlated with high costs, that are economically unattractive. Bearing this in mind, magnetic adsorbents, especially those in the nanoscale size range, can seem to be a great solution. Adsorbents’ magnetic response can serve as a simple and costless separation process, whereas nano-size and large specific surface area enables more active adsorption sites for HM removal. Such magnetic adsorbents based on magnetic iron-oxide (MNPs) are environmentally friendly, costless, and can be produced on a large scale [8,13]. Despite their advantages, bare/unmodified MNPs seem to be less efficient in the adsorption process, as the bare MNPs` surface is relatively inert [14]. Therefore, they may be coated with a thick silica layer that improves the density of the –OH groups significantly, and can be further employed for chemical linkage with additional substrates [15]. Moreover, silica porosity increases surface area, it is chemically stable, costless, and shields the bare MNPs from dissolution in acidic aqueous media [16]. However, the thickness of the silica layer should be limited, because it dilutes the saturation magnetization [15].

The amino-(bio)polymers with nitrogen-donor functional groups can be used for chelation with HMs [17]. Such polymer, with amino-rich functional groups, is branched polyethyleneimine (bPEI), consisting of primary, secondary and ternary amines accompanied by highly hyper-branched structure [18]. Because of its solubility in an aqueous environment, it is often immobilized on the particle/support. The combination of the latter has been shown to be not only a biocompatible material in biomedical applications [19], but also in HM removal applications [20,21,22], together with anionic dyes and disinfection [23,24,25]. Alternatively, due to the high density of functional groups (i.e., primary amines), bPEI can be crosslinked to an organic functional reactive group, such as the epoxy group. Covalent coupling to polymeric particles possessing primary amine functionalities at the polymers` backbone through reaction with the epoxy-organosilanes containing groups, results in secondary amine and ring-opening reaction facilitated under alkaline conditions. One of the very useful organosilane modification agents is 3-glycidoxypropyltrimethoxysilane (GOPTS). GOPTS carries reactive epoxy groups on one side of the molecule, and a silane group on the other side. Inorganic surfaces coated with an amorphous silica layer can be used for covalent linkage with amine-containing ligands at specific pH of the reaction. In this context, GOPTS can be used to link the inorganic silica (terminated with abundant –OH groups) with the ligand, having a primary amine group [26].

It is generally known that metal adsorption efficiency is highly dependent on good magnetic response, large surface-to-volume ratio, many active bonding sites, and the irreversibly linked environmentally-friendly adsorbent on a magnetic carrier [8,13]. Taking this into consideration, the MNPs coated with a thick silica layer and chemically modified with bPEI, could represent an ideal adsorbent. Recent studies attached bPEI onto MNPs via electrostatic and/or physical interactions [21,27,28,29], causing unstable coatings. Furthermore, hazardous chemicals were used [30], or the process included several synthesis steps to achieve the desired product, being rather unattractive economically [20,24,31]. Regarding bPEI-based magnetic adsorbent, studies were focused mainly on the removal of chromium [31,32,33] or lead [22,28,30], whereas such kinds of polyethyleneimine-derived silica-coated MNPs for copper removal were not found in the relevant literature. Although a similar study for linkage of MNPs@SiO_2_ with bPEI was recently reported [34], their synthesis concept and methodology differ significantly from our study. Moreover, the designed micro-scale adsorbent was studied for uranium removal applications.

Surprisingly, to the best of our knowledge, no detailed and systematic study regarding the crosslinking of GOPTS with bPEI and its further silanization directly onto core-shell silica-coated MNPs for Cu^2+^ removal has been reported yet. There are several advantages of the introduced amino-based nanoadsorbents with respect to current state-of-the-art adsorbents: (i) Environmentally-friendly adsorbent, synthesized easily with the concept of green chemistry; (ii) The combination of the amino-biopolymer together with silica-modified MNPs ensures more functional sites for covalent bonding, easy manoeuvrability and removal of the adsorbent with no need for additional operating costs; (iii) Strongly covalently attached GOPTS-modified bPEI onto silica-coated MNPs ensures a strongly, permanently bonded amino-polymer that does not desorb during the cleaning procedure, and, consequently, improves adsorption efficiency, (iv) Systematic and detailed characterization of the physicochemical properties in terms of surface chemistry, charge, morphology, ligand density, etc. and (v) Plenty of primary amine groups from the bPEI macromolecule backbone that can serve as an HM chelator. An important, but often overlooked aspect, is that the efficient adsorption ability, together with quick magnetic response, should also be considered.

This work represents a comprehensive study of chemically modified bPEI with an epoxy group, and its silanization onto core-shell MNPs@SiO_2_ for copper removal application. The synthesis parameters (silica shell thickness, amount of derived-bPEI and pH) were studied in detail, and accompanied by extensive nanoadsorbent characterization. The adsorption capacity was studied with Cu^2+^ aqueous model solutions to define the optimal parameters for transfer into real applications. The results revealed superior MNPs@SiO_2_@GOPTS-bPEI ability for Cu^2+^ removal due to the high density of amino groups. Moreover, adsorption studies were performed analogously for MNPs@SiO_2_, emphasizing the importance of the introduced amino-biopolymer on removal efficiency. Additionally, the mechanism of copper adsorption was proposed, employing different surface analysis techniques.

## 2. Materials and Methods 

### 2.1. Materials and Chemicals:

FeSO_4_·7H_2_O was purchased from Honeywell (Seelze, Germany). Fe_2_(SO_4_)_3_·7H_2_O, HCl (≥37%), tetraethylorthosilicate (TEOS, ≥98%) and branched PEI with average Molecular Weight (MW) of 25,000 Da, copper(II) chloride (97%) and 3-glycidoxypropyltrimethoxysilane (GOPTS, 98%) were all purchased from Sigma-Aldrich (Taufkirchen, Germany). NH_4_OH (25% aqueous solution), NaOH (>98%) and acetone (≥99.5%) were purchased from Honeywell (Seelze, Germany). Absolute EtOH (anhydrous) was obtained from CarloErba (Val de Reuil, France), and citric acid (≥99.5%, water free) from Roth (Karlsruhe, Germany). All chemicals were used as received, without any further purification. Ultrapure water (with a resistivity of 18.2 MΩ cm, obtained from Milli-Q, Millipore Corporation, MA, USA) was used throughout the experiments.

### 2.2. Preparation of γ-Fe_2_O_3_ (MNPs) and SiO_2_-coated MNPs (MNPs@SiO_2_)

Magnetic nanoparticles based on maghemite, abbreviated hereinafter as MNPs, were synthesized under air atmosphere with coprecipitation [35]. Fe^2+^ and Fe^3+^ aqueous solution (*V* = 500 mL) with the *n*(Fe^2+^):*n*(Fe^3+^) = 2.4:1 ratio was prepared, using iron sulphate reagents, salts and ultrapure water. In order to precipitate iron hydroxides, a diluted aqueous ammonia solution was added slowly to an iron salts solution at pH 3. For precipitation and subsequent formation of magnetic iron oxide NPs, 250 mL of ammonia solution (25%) was added to the above mixture and agitated additionally for 30 min. Synthesized MNPs were washed several times with the diluted ammonia solution and ultrapure water. Afterwards, the colloidal stable dispersion of MNPs was prepared using the adsorption of Citric Acid (CA) in accordance to Reference [35]. Approximately 1.2 g of as-synthesized bare MNPs were redispersed in 60 mL of ultrapure water, together with the addition of 5 mL (0.5 g·mL^−1^) CA aqueous solution. Dispersion pH was raised to 5.2 with the diluted ammonia solution, and put under reflux for 1.5 h at 80 °C. Following refluxing, the pH of the cooled dispersion was set to ~10 with ammonia solution (25%). The coating of MNPs stabilized with CA with silica shell (MNPs@SiO_2_) was applied in similar way as in Reference [15], by introducing some modifications to achieve a 3 nm thick silica layer. NH_4_OH was added to the MNPs@CA dispersion (15 mg·mL^−1^, pH = 10.6). The mixture was agitated for 15 min and added rapidly to the solution of EtOH and TEOS (10 mg·mL^−1^). This was followed with pH settling to 10.6, using 25% NH_4_OH. The coating procedure was left to proceed for 2 h under continuous stirring. The obtained core-shell MNPs@SiO_2_ were cleaned of the excess reagents using absolute EtOH and ultrapure water.

### 2.3. Systematic Covalent Linkage of GOPTS and bPEI (GOPTS-bPEI) onto MNPs@SiO_2_ (i.e., MNPs@SiO_2_@GOPTS-bPEI)

**Scheme 1:**
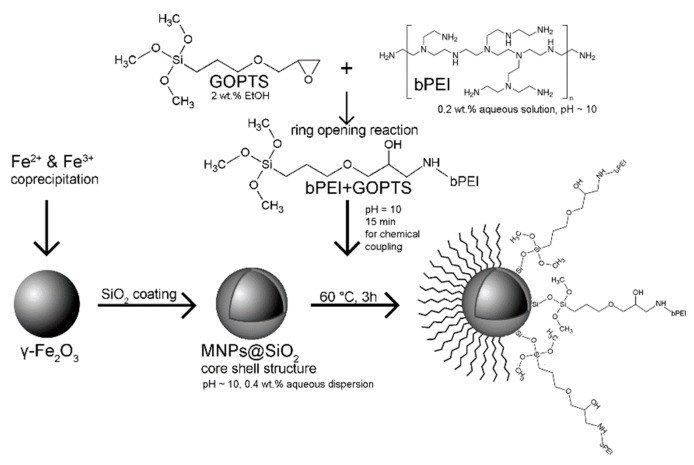
Proposed chemical coupling mechanism for MNPs@SiO_2_@GOPTS-bPEI.

In order to perform synthesis systematically, some calculations were performed to avoid the excess of reagents, that can influence the MNPs@SiO_2_ stability significantly during the modification process and decrease the silanization efficiency. The theoretical specific surface area of MNPs@SiO_2_ was estimated to be around 95 m^2^_·_g^−1^, and the calculation was done in accordance with Čampelj et al. [35]. The estimated surface concentration of the Si-OH groups is 5 per 1 nm^2^ [16], where each silanol group will hydrolyse with the methoxy-group from GOPTS. On the other hand, it was predicted that one epoxy ring will react with one primary amino group from bPEI (Scheme 1). Based on these predictions, the reaction conditions for synthesizing the MNPs@SiO_2_@GOPTS-bPEI were as follows. For functionalization of 100 mg of MNPs@SiO_2_ with GOPTS-bPEI, bPEI was dissolved in deionized water (*V* = 20 mL, 0.2 wt.%, bPEI value added to MNPs@SiO_2_ corresponded to five monomers bPEI per 1 nm^2^ of MNPs@SiO_2_). pH was adjusted to 10 with 0.1 M HCl. Simultaneously, GOPTS (5 molecules per 1 nm^2^ of MNPs@SiO_2_) was dissolved in absolute EtOH (2 wt.%). After preparation of both solutions, the solution of GOPTS was added slowly to the bPEI aqueous solution, with the pH remaining at 10. It is widely accepted that the amine nucleophiles react with epoxy functionalities at moderate alkaline area (at least pH = 9) [26]. The mixture was left to stir for 15 min, and resulted in a clear solution with the absence of aggregates. The formed chemically coupled GOPTS-bPEI was then added to 0.4 wt.% MNPs@SiO_2_ aqueous dispersion at pH = 10, where repulsive negative forces among the MNPs@SiO_2_ should be strong enough to enable stable dispersion during the functionalization. Silanization reaction was left to proceed for 3 h under reflux at 60 °C. The proposed chemical coupling mechanism is shown schematically in Scheme 1. After chemical linkage, the dispersion was separated with a magnet and washed with acidic ultrapure water (pH = 4, adjusted with 0.1 M HCl) several times.

### 2.4. Characterization of the Nanoadsorbent

The crystal structure and purity of as-synthesized bare MNPs was verified with X-Ray powder Diffraction (XRD) using a D-5005 diffractometer Bruker Siemens with CuKα radiation, *λ*_CuKα_ = 1.5406 Å. Parameters of the XRD measurements were 2θ from 30 to 70°, and the scan rate was 0.385° min^−1^. Morphology, and nanoscale particle size of the bare and core-shell structure of coated MNPs were observed with Transmission Electron Microscopy (TEM, Jeol JEM-2100), operating at the acceleration voltage of 200 kV. For TEM investigations, dispersions were deposited onto a copper grid with transparent carbon film. Specific surface area, as well as equivalent diameter of particles, were estimated and calculated from the TEM images using Gatan Digital Micrograph Software. The Scanning Electron Microscope (Carl Zeiss FE-SEM SUPRA 35 VP) was used for the observation of the nanoadsorbent morphology. The nanoadsorbent was dried, put onto a double-sidde adhesive conductive carbon tape, placed onto an aluminium sample holder, and observed at an acceleration voltage of 1 keV, 30 μm-sized aperture, 5.5 mm working distance, and at 100,000× magnification. The surface modification steps were inspected with infrared spectroscopy (FTIR, PerkinElmer Spectrum GX spectrometer equipped with a diamond crystal Attenuated Total Reflection (ATR) attachment). For the latter, samples were dried, placed on a diamond crystal, and pressed into the thick film. Background, as well as all spectra, were reported as an average of 32 scans, that were recorded from 400–4000 cm^–1^ with a resolution of 2 cm^–1^ at room temperature. The weight fraction of mass loss was determined via thermogravimetric analysis (TGA/SDTA 851 Mettler Toledo). Dry samples were placed into 60 μL alumina crucibles and heated from 25 °C to 600 °C with a rate of 10 K·min^−1^ in an O_2_ atmosphere. Zeta Potential (ZP) and hydrodynamic diameter, with corresponding number-sized distribution in aqueous dispersions, were monitored with the electrokinetic measurements via Dynamic Light Scattering by the electrophoresis experiments of the samples (DLS ZetaSizer Nano ZS, Malvern Instruments Ltd). For ZP measurements in aqueous dispersions, disposable folded capillary cell having electrodes was filled up and placed into a sample holder. Similarly, for the hydrodynamic diameter, the disposable cuvette was filled up (to 1 cm) with the aqueous dispersion. Particular pH measurement was adjusted with 0.1 M HCl or 0.1 M NaOH in 1 mM NaCl background at a constant temperature of 25 °C. At the end, the measurement data were collected with the usage of the manufacturer`s software. pH-dependent potentiometric titrations were performed for the total charge determination, as well as for pK values` determination of the pure bPEI solution. The latter was carried out in a forward (acidic to alkaline) and backward direction (alkaline to acidic) at 2.5 < pH < 11.0, using 0.1 M HCl and 0.1 M KOH aqueous solutions as titrants. A twin burette instrument (Mettler T-70) was equipped with a pH electrode (Mettler Toledo InLab Reach 225). All solutions for potentiometric titrations were prepared with ultrapure water having a very low carbonate content (<10^−6^ M), and the ionic strength adjusted to 0.1 M with 3 M KCl. The blank titrations were performed under the same conditions as depicted above. A detailed description regarding the charge calculations and *p*K value determination can be found in Reference [36]. Room-temperature magnetization curves of samples were measured with a Vibrating-Sample Magnetometer (VSM, Lake Shore 7307). X-ray Photoelectron Spectroscopy (XPS) measurements were performed using a PHI-TFA 5600 XPS spectrometer from Physical Electronics Inc., which was equipped with a monochromatic Al X-ray excitation source (photon energy of 1486.6 eV) and a hemispherical electron analyzer. Spectra were acquired at 45° take-off angle. The radius of the analyzed area was 0.4 mm. An additional electron gun was used to compensate for the possible charging effect of the samples. Survey and high-resolution C 1s, N 1s, Si 2p and Fe 2p spectra were measured. The analysis of XPS spectra was performed with Multipak 8.1c software. A C 1s high-resolution spectrum was employed at a binding energy of 284.8 eV to correct the binding energy scale. The surface area and the total pore volume were determined with the adsorption–desorption of N_2_ at 77 K, and analyzed further employing a BET analyzer (Micromeritics TriStar II Surface Area and Porosity), whereas results were subjected and analyzed with the TriStar II 3020 version 3.02 software package. To assure pure nanocomposites and the removal of any adsorbed contaminants, samples were degassed prior to surface area analysis. 

### 2.5. Cu^2+^ Adsorption Experiments

A Cu^2+^ stock solution (5 g·L^−1^) was prepared from CuCl_2_. It is known that Cu^2+^ adsorbs [21] onto bPEI selectively, while the Cl^−^ was chosen as an anion, because the importance of an appropriate anion with respect on removal efficiency [37] has already been shown. Solutions for further experiments were prepared by dilution of the stock solution to the defined concentration (5−150 mg·L^−1^, i.e., initial Cu^2+^ concentrations were 5, 10, 25, 50, 75, 100, and 150 mg·L^−1^). The batch adsorption experiments were performed in 150 mL flasks under vigorous magnetic stirring. The 5 mg of nanoadsorbents were placed together with the 50 mL of Cu^2+^ solution in the flask at constant temperature (298 K), t = 1 h. The specific pH (3−6) was adjusted with the use of 0.1 M NaOH or HCl. After the adsorption process, the magnetic nanoadsorbent was removed immediately with the permanent magnet. Just to assure the elimination of any remaining nanoadsorbent, the supernatant was filtered through an MW 10 kDa filter using ultrafiltration. The supernatant was diluted with ultrapure water, and its quantity of residual Cu^2+^ was determined with Atomic Absorption Spectroscopy (AAS). AAS was employed for determination of the Cu^2+^ in supernatants after the adsorption process using a Perking Elmer 3110 spectrophotometer. Prior to analysis, the calibration curve of Cu^2+^ was prepared, followed by measurements of Cu^2+^ in solutions at a wavelength of 324.8 nm. Each measurement was done in triplicate, and the average results are reported. The Standard Deviation with this analytical technique was calculated to be 10% of the measured value. The adsorption capacity (*q*) and removal efficiency (*R*) were calculated with the following equations:

*q* (mg·g^−1^) = (*c*_0_ − *c*_t_)*V*/*m* (1)

*R* (%) = (1 − *c*_t_/*c*_0_) × 100 (2)

*q* is the adsorption capacity (mg Cu^2+^ per g of the adsorbent), *R* is the removal efficiency, *c*_0_ is the initial Cu^2+^ concentration in solution, and the *c*_t_ is the Cu^2+^ concentration in the supernatant after treatment, *m* is the mass of the nanoadsorbent (g), and *V* is the volume of the Cu^2+^ model solution.

Reusability cycles by means of adsorption–desorption experiments were performed with MNPs@SiO_2_@GOPTS-bPEI by batch experiment, similar as already explained above. After finished adsorption, the magnetic nanoadsorbents were decanted onto the permanent magnet. For the following desorption studies, the nanoadsorbents with Cu were immersed in 10 mL of 0.1 M Na_2_EDTA and left to agitate for 2 h. The Na_2_EDTA was selected as an eluent, due to its known exceptional desorption capability [38]. Finally, MNPs@SiO_2_@GOPTS-bPEI with desorbed Cu were again collected onto the permanent magnet, rinsed with ultrapure water, and applied further in the next reusability cycle.

## 3. Results and Discussion

### 3.1. Nanoadsorbent Characterization

**Figure 1:**
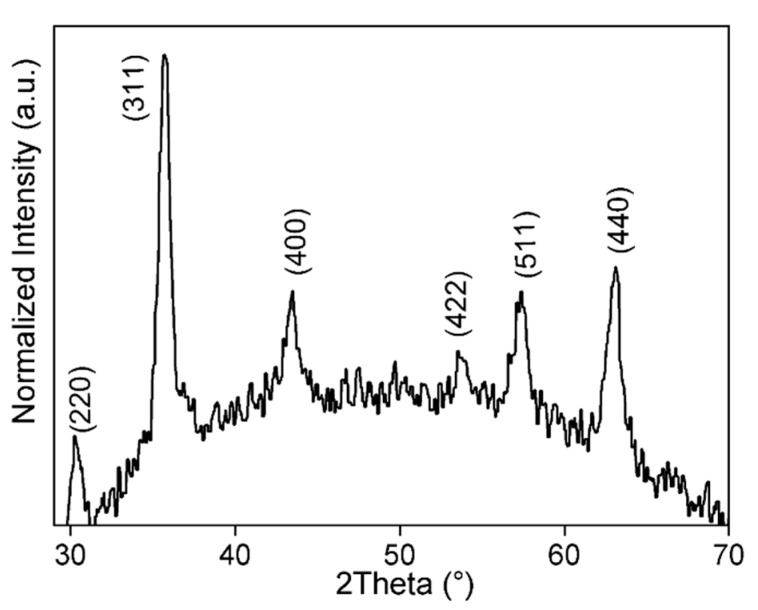
XRD pattern for synthesized bare magnetic nanoparticles (MNPs.)

The crystal structure of the magnetic part was verified with XRD analysis (Figure 1). The results revealed that the MNPs crystal structure was assigned to maghemite (Figure 1), as diffraction peaks corresponded to a standard reference card (JCPDS 72-0246, cubic space group Fd-3m), typical for a maghemite cubic spinel crystal structure. No other peaks were observed, indicating the phase purity of the synthesized bare MNPs. Additionally, broadening of the diffraction peaks shows the nanocrystallinity of the synthesized MNPs. In the continuation, regardless of the surface modification, the crystal structure remained unchanged.

**Figure 2:**
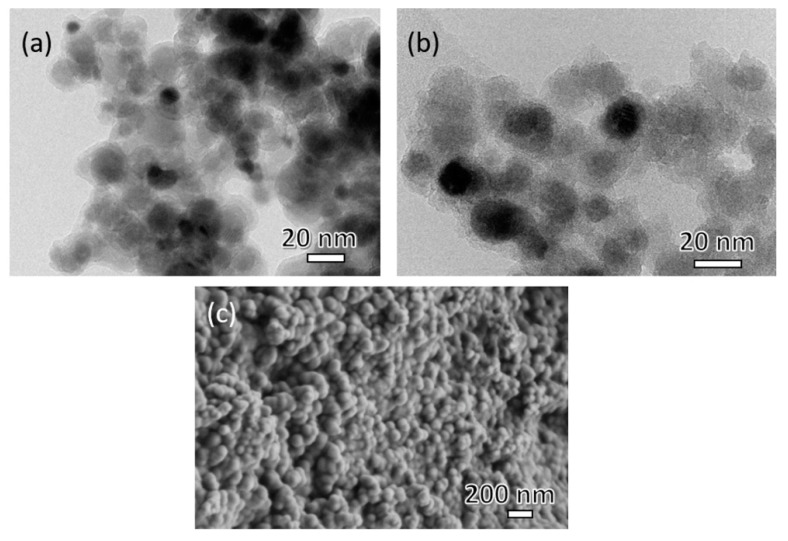
TEM image of MNPs@SiO_2_ (**a**), TEM image of MNPs@SiO_2_@GOPTS-bPEI (**b**), and representative SEM image of MNPs@SiO_2_@GOPTS-bPEI (**c**). Note that darker spots in the TEM images correspond to the bare MNPs that overlap when deposited onto the copper grid.

The morphology and the shape of the silica-coated MNPs before and after functionalization with GOPTS-bPEI were investigated with TEM and SEM. Representative images are shown in Figure 2. It can be seen clearly that the MNPs (~13 nm) were of quasi-spherical shape, and were coated uniformly with around a 3 nm thick silica layer. The silica layer is surrounding the MNPs, which results in a core-shell structure (Figure 2a,b). The silica layer is amorphous, and it is seen in a higher contrast (gray) with respect to the core–magnetic part (dark). The optimal synthesis conditions reassured that almost all observed MNPs@SiO_2_ had similar SiO_2_-shell thickness with any observation of the homogeneously formed SiO_2_ NPs. After the functionalization of MNPs@SiO_2_ with the GOPTS-bPEI, no obvious influence on the morphology was observed with the additional silanization step (Figure 2b). We can conclude that grafting affects neither the morphology, nor the silica-shell thickness of the nanoadsorbent. A more clear morphology of the introduced MNPs@SiO_2_@GOPTS-bPEI can be revealed from the SEM image shown in Figure 2c. The latter shows a quasi-spherical morphology that is in agreement with the TEM images, however, with the larger agglomeration degree that can be related to the difference of magnetic nanocomposite sample preparation for observation. Nevertheless, from Figure 2c, individual MNPs@SiO_2_@GOPTS-bPEIs can also be distinguished, but at the lower magnification, as in the case of the TEM images.

**Figure 3:**
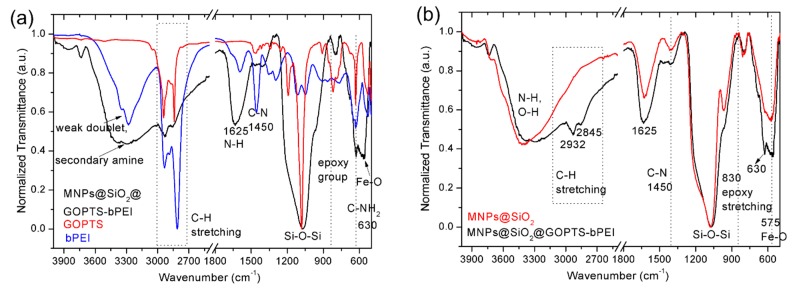
FTIR spectra of MNPs@SiO_2_@GOPTS-bPEI with respect to pure bPEI and GOPTS (**a**). FTIR spectra of MNPs@SiO_2_ and MNPs@SiO_2_@GOPTS-bPEI (**b**).

The surface ATR-FTIR spectroscopy analysis confirmed the successful introduction of coatings (Figure 3). FTIR spectra of synthesized nanoadsorbent MNPs@SiO_2_@GOPTS-bPEI with respect to the pure GOPTS and bPEI are shown in Figure 3a. The intensive band at 575 cm^−1^ originates from the magnetic part of the MNPs, and corresponds to the Fe–O vibrations of Fe_2_O_3_ [14]. The peak at around 1090 cm^−1^ corresponds to the Si–O–Si asymmetric stretching vibrations, representing the presence of SiO_2_ coating in MNPs@SiO_2_ [39]. Furthermore, the peaks at 2932 and 2845 cm^−1^, ascribed to asymmetric stretching of CH_2_ and symmetric stretching of CH groups, are evidently seen at MNPs@SiO_2_@GOPTS-bPEI, confirming the presence of bPEI [40]. Moreover, the spectrum for pure bPEI shows a weak doublet at high frequencies, corresponding to primary amine [41]. After reaction of GOPTS with bPEI and its introduction onto MNPs@SiO_2_, the latter is evidently not seen anymore. This can be correlated with the ring-opening process between the nucleophilic primary amine group of bPEI and epoxy group of GOPTS, which results in secondary amine formation (Scheme 1). Meanwhile, the epoxy bands vibrations located about 1270 and 830 cm^−1^ decreased significantly, indicating a ring-opening process and confirming our bonding mechanism between bPEI and GOPTS. To provide further information about efficient surface modification, the FTIR spectra of MNPs@SiO_2_ was compared to the MNPs@SiO_2_@GOPTS-bPEI (Figure 3b). The presence of peaks from the MNPs@SiO_2_ core-shell nanocomposite, together with the newly introduced vibrational bands from GOPTS-bPEI, confirms the successful crosslinking. Specifically, the band at 627 cm^−1^ corresponding to the C-NH_2_ vibrations, band at 1450 cm^−1^ attributed to C–N groups from bPEI and C–H stretching at 2932 and 2845 cm^−1^ [41], show the successful functionalization clearly.

**Figure 4:**
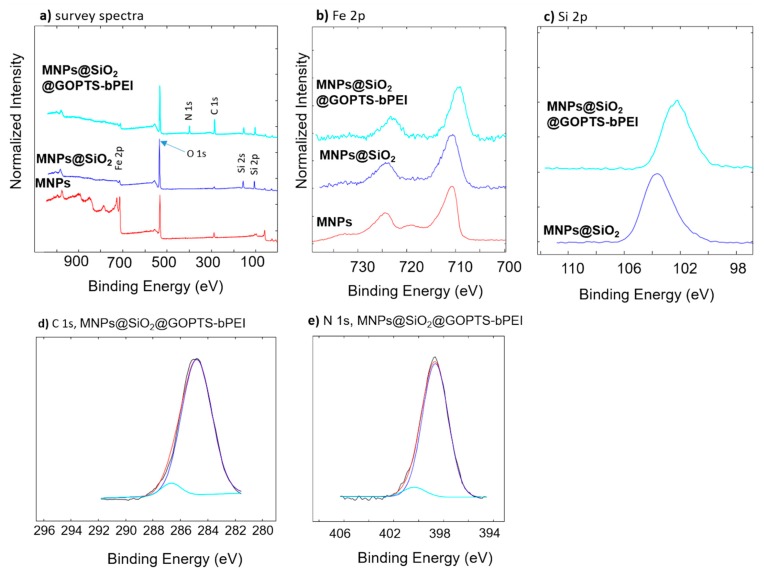
(**a**) Survey spectra and high-resolution, (**b**) Fe 2p and **(c**) Si 2p spectra. Deconvoluted high-resolution, (**d**) C 1s spectrum for MNPs@SiO_2_@GOPTS-bPEI, and (**e**) N 1s spectrum MNPs@SiO_2_@GOPTS-bPEI.

The XPS technique is one of the most powerful surface sensitive techniques, and was employed in this study for the determination of the samples’ surface atomic composition. The determined atomic concentrations for MNPs, MNPs@SiO_2_, MNPs@SiO_2_@GOPTS-bPEI are reported in Table 1. Corresponding survey and high-resolution spectra are shown in Figure 4.

The atomic concentration ratio of Fe:O for MNPs is in good agreement with the stoichiometric ratio of MNPs, and confirms Fe_2_O_3_–Fe (31.4 at.%) and O (53.2 at.%) (Table 1). A C signal was detected along with Fe and O. The latter is most likely a consequence of the adventitious carbonaceous species` adsorption (compounds that are present in the atmosphere) on the MNPs` surface during the preparation procedure [42]. Moreover, in agreement with the XRD results, the high-resolution Fe 2p spectra show an intensive peak at the binding energy of 710.9 eV (Figure 4b), confirming the Fe_2_O_3_ structure [43].

As expected, by further surface modification of MNPs with the amorphous silica layer, the Si signal was detected (MNPs@SiO_2_, Figure 4a,c). Consequently, the Fe atomic concentration decreased significantly on the MNPs` surface (Table 1), due to the presence of the SiO_2_ surface coating. The analyzed depth in the XPS studies is calculated as 3*λ*sin*θ* (an estimation where about 90% of the XPS signal originates), where *λ* stands for the inelastic mean free path, and *θ* is the take-off angle (in the present case 45°). Therefore, as Fe was still detected, and due to the fact that the analyzed depth in this case is about 5 nm, the thickness of the SiO_2_ is thinner than 5 nm. In fact, using TEM, it was determined to be around 3 nm (Figure 2a,b). High-resolution Si 2p spectra confirm that the source of Si originates from the SiO_2,_ as the peak is located at the binding energy of 103.5 eV (Figure 4c). Moreover, the SiO_2_ surface coating on MNPs did not change the shape and peaks` position of the Fe 2p spectrum compared with that for MNPs, indicating that the surface coating with SiO_2_ on the Fe_2_O_3_ structure did not modify the Fe_2_O_3_ (Figure 4b).

Followed by further functionalization of MNPs@SiO_2_ with the chemically linked GOPTS−bPEI, the Fe signal is still detected (1.9 wt.%). However, the amount is lower than that for the MNPs@SiO_2,_ due to the even thicker surface coating regarding the additional layer. Additionally, the Si amount decreased to 14.6 at.%. On the contrary, with the MNPs@SiO_2_ modification, the N signal was detected (9.1 at.%), which confirms the presence of bPEI. Moreover, the amount of the C also increased, as C atoms are the main constituents in the hyper-polymer branched structure. After the surface modification of the MNPs@SiO_2_ with the GOPTS-bPEI, the Si 2p peak was transferred to the more negative binding energies (102.2 eV, Figure 4c) that can be attributed to the silanization process. The latter is in high agreement with our proposed bonding strategy (Scheme 1). The deconvoluted C 1s spectrum for MNPs@SiO_2_@GOPTS-bPEI is shown in Figure 4d. The latter was fitted with two peaks, i.e., a feature at 284.8 eV corresponding to C–C/C–H bonds in the polymer backbone, and a feature at 286.7 eV that corresponds to C–O and/or C–N [44]. The presence of C–O species definitely originates from the cross-linked GOPTS-bPEI via a ring-opening reaction (Scheme 1), resulting in ether formation. On the other hand, C–N originates from the bounded bPEI. The deconvolution of the high-resolution N 1s spectrum for MNPs@SiO_2_@GOPTS-bPEI showed that the major contribution corresponds to the C–N species in bPEI, while the deconvoluted high binding energy peak can be attributed to the small amount of protonated amino groups in the bPEI (Figure 4e) [43].

**Figure 5:**
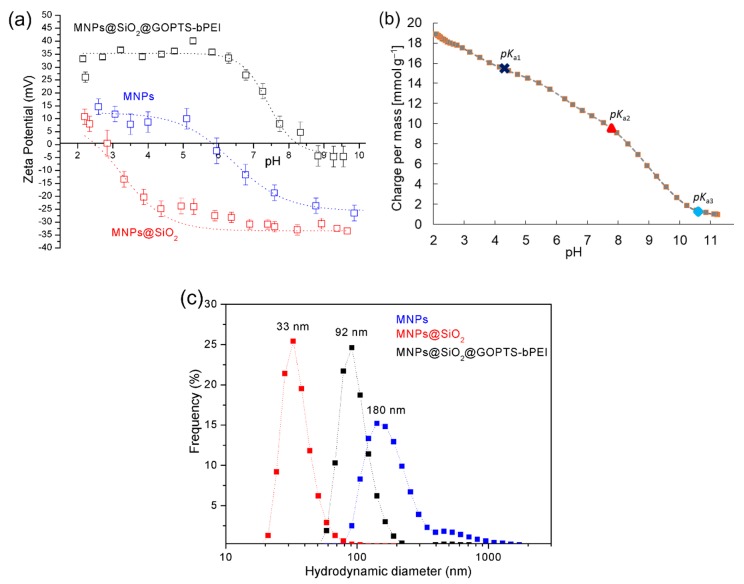
Zeta Potential measurement as a function of pH for SiO_2_-coated MNPs and the same one functionalized with the GOPTS-bPEI (**a**); pH-depended potentiometric titration versus pH for pure hyper-branched polyethyleneimine (bPEI) (**b**) and number-sized distribution of hydrodynamic diameter at pH = 6 in aqueous media (**c**).

Surface charge as a protonation/deprotonation ratio of functional groups is changing as a consequence of different modifications steps, and was correlated with the electrokinetic measurements. The latest results were supported with Dynamic Light Scattering. The comparison of Zeta Potential (ZP) values versus pH for MNPs, MNPs@SiO_2_ and MNPs@SiO_2_@GOPTS-bPEI are shown in Figure 5a. After coating of MNPs with an amorphous silica layer, SiO_2_-coated MNPs expressed negative ZP values in almost the entire pH range (isoelectric point (IEP) at pH ~2.5) due to the presence of negatively charged silanol groups from the silica coating. With further GOPTS-bPEI functionalization to MNPs@SiO_2_, the IEP shifted into an alkaline area (Figure 5a). At pH < 8.5, the amino groups were protonated, resulting in a positive charge [21], originating from the amino groups onto the bPEI-functionalized MNPs@SiO_2_ surface. In contrast, at pH > 8.5, the amino groups at the MNPs@SiO_2_@GOPTS-bPEI became deprotonated. As can be seen from Figure 5a, the shift of GOPTS-bPEI-functionalized MNPs@SiO_2_ IEP to a higher pH value (pH_IEP_ ≈ 8.5), which also confirms the successful covalent bonding of GOPTS-bPEI onto MNPs@SiO_2_. Results are in high agreement with the pH-dependent potentiometric charge titration. It can be observed that the pure bPEI protonates/deprotonates in 3-pK fashion that lies between the plateaus at pH =3 and pH = 11 (Figure 5b). This is due to the contribution of the amine groups, which exhibit a highly positive charge (~15.7 mmol·g^−1^). As the bPEI is crosslinked with the GOPTS and functionalized onto MNPs@SiO_2_ (Scheme 1), the pH_IEP_ ≈ 8.5 is near to the *pK*_a2_ value of pure bPEI for the secondary amine, being around 7.8 (Figure 5b), which again proved the successful chemical coupling as proposed in Scheme 1. This phenomenon also supports the fact of the pure bPEI being in almost deprotonated state, expressing its high nucleophilicity when cross-linked to GOPTS at pH 10 (for details see the Experimental Section).

Dynamic Light Scattering was employed for estimation of the hydrodynamic diameter and size distribution when the nanoadsorbent was exposed to the aqueous environment. The latter were measured at pH = 6 (close to neutral range) for both aqueous dispersions (Figure 5c), where they exhibited the largest absolute ZP values according to the ZP measurements (Figure 5a). The hydrodynamic diameter of MNPs (~180 nm) is obviously larger in comparison to the MNPs@SiO_2_ (~33 nm). This can be attributed to the high amount of anionic charged silanol groups from the MNPs@SiO_2,_ that provided high electrostatic repulsion and, thus, overcame the agglomeration. After the functionalization with GOPTS-bPEI, the hydrodynamic diameter increased (~92 nm), but the narrow size distribution is still observed. This fact correlated well with the highly positive nature of amine groups (i.e., secondary and ternary amine groups) being protonated and the hyper-branched structure of bPEI. Synergistically, both effects reassured the electrosteric repulsion. For the application of the nanoadsorbent, the nanosize and its distribution in aqueous media plays a crucial role in the achievement of better removal efficiency.

**Figure 6:**
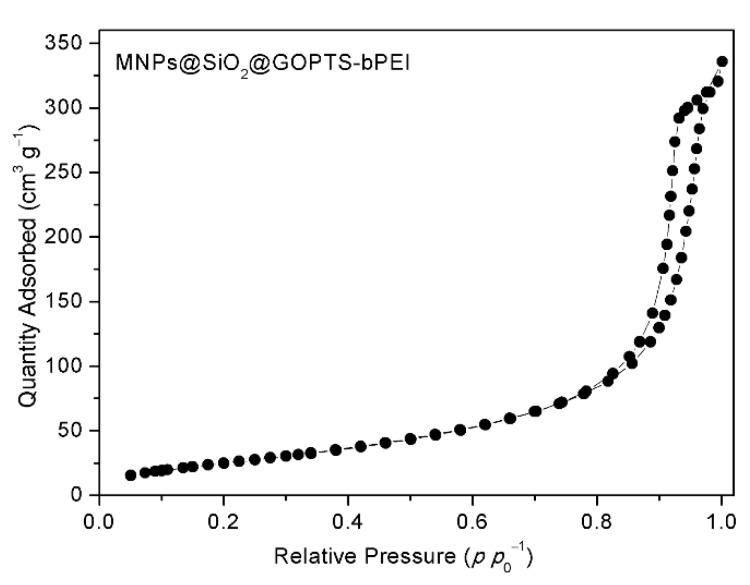
Graph of N_2_ adsorption and desorption isotherm (77 K) as a function of relative pressure of MNPs@SiO_2_@GOPTS-bPEI.

The synthesized magnetic nanocomposite was also characterized by means of the surface area and pore volume with the Brunauer–Elmett–Teller (BET) theory and Barett–Joyner–Halenda (BJH) method, respectively. It can be observed from Figure 6, that the adsorption–desorption isotherm graph of N_2_ exhibits the typical IV type BET isotherm for MNPs@SiO_2_@GOPTS-bPEI, and this phenomenon is attributed mainly to its mesoporous structure [45]. Quite a high specific area was measured for MNPs@SiO_2_ (25.21 m^2^·g^−1^), and the latter is significantly increased with respect to the bare MNPs (for 86% larger surface area, Table 2) with introducing only a 3 nm thick layer. With additional surface modification using GOPTS-bPEI, the surface area increased to 38.33 m^2^·g^−1^ (52% increase) in comparison to the MNPs@SiO_2_. Following the same trend, the total pore volume increased from 0.16 cm^3^·g^−1^ to 0.23 cm^3^·g^−1^ with additional functionalization (Table 2), and is in contrast to the reports [17,46], where they observed a decreased porous volume and surface area after functionalization with bPEI. This fact can be correlated to the modified MNPs@SiO_2_ surface with GOPTS-bPEI that most probably causes the formation of additional intraparticle space, or pores originating from the derived polymer network structure. Indeed, the results indicate an even larger surface area and pore volume when modifying MNPs@SiO_2_ surface by GOPTS-bPEI, showing an enlarged effective adsorption area towards successful HM removal.

**Figure 7:**
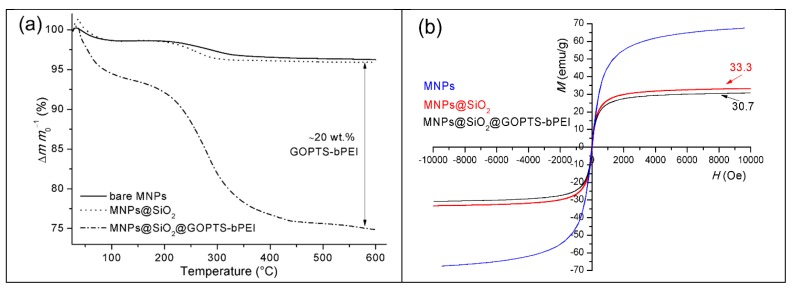
TGA curves for MNPs, MNPs@SiO_2_ and MNPs@SiO_2_@GOPTS-bPEI (**a**). Room-temperature hysteresis loops for bare MNPs, SiO_2_-coated MNPs and MNPs@SiO_2_@GOPTS-bPEI (**b**).

Overall, the thermal decomposition of the introduced bPEI-derivate as a function of temperature was measured with TGA, and ensured the weight mass loss regarding the GOPTS-crosslinked bPEI. The TGA profiles for bare MNPs, MNPs@SiO_2_ and MNPs@SiO_2_@GOPTS-bPEI are shown in Figure 7a. Bare MNPs display small mass loss (3 wt.%), most likely due to the physisorbed water or residuals from the solvents. A small increase in mass loss was measured for MNPs@SiO_2_ (5 wt.%), and can be correlated to the presence of a higher density of –OH groups that release from the silica coating during heating. A very different story can be seen for MNPs@SiO_2_@GOPTS-bPEI. The first step in weight loss occurred at around 100 °C, similar as in the case of MNPs@SiO_2,_ indicating the mass loss due to –OH release. With further heating, the mass loss increased significantly, indicating the GOPTS-bPEI degradation into volatile fragments, and the latter decomposed completely at *T* = 500 °C. The weight loss regarding the GOPTS-bPEI was estimated to be around 20 wt.%, and is associated with the polymers` thermal degradation into volatile products [44]. With the combining mass loss regarding the GOPTS-bPEI, a rough estimation of the ligand number density per nm^2^ can be calculated, assuming that all the excess of reagents was removed by washing and magnetic decantation. Similar ways of ligand density calculations were reported, and the details can be found elsewhere [47]. The value of silanol groups was already reported in literature, and was estimated to be around 5 SiOH nm^−2^ [16]. Ligand density, together with weight loss, accounts to be three molecules of GOPTS-bPEI nm^−2^. If the assumption of the one primary amino group linkage to the epoxy group is expected (Scheme 1), then three primary amino groups (at pH = 10 full deprotonation of primary amines is expected; refer to the experimental part) could be used for metal-ion chelation. In this context, the density of –NH_2_ groups per nm^2^ can be estimated to be 9 NH_2_ nm^−2^. We can definitely sum up that synthesized nanocomposite exhibiting the surface with amino-rich groups is a promising sorbent for HM via chelation.

The magnetism of bare and differently surface-modified MNPs was explored by room-temperature magnetization curves (Figure 7b). After the coating of the MNPs with a 3 nm thick silica layer, the saturation magnetization decreased from 68 emu·g^−1^ to 33.3 emu·g^−1^ (Figure 7b, plateau part). The dependency of magnetic properties of the amount of the incorporated magnetic phase, shape and size is widely known [14]. Consequently, in nanocomposites constituted from the nonmagnetic and magnetic phases, the amount of incorporated magnetic phase amount plays an important role. In this way, the presence of a nonmagnetic component such as a silica layer reduces the saturated magnetization. With further functionalization of MNPs@SiO_2_ with the GOPTS-bPEI, the saturated magnetization of the MNPs@SiO_2_@GOPTS-bPEI decreased to 30.7 emu·g^−1^, due to the additional presence of the nonmagnetic phase originating from the covalently linked amino-rich polymer as an additional layer surrounding the SiO_2_-coated MNPs. Among reported values correlated to the saturated magnetization, the reported value herein is one of the largest, just to compare with a few [29,31,48] based on magnetic-bPEI, and surely expresses enough high magnetic force to be applied in real metal-removal application with the synergetic effect of the high density of amino-chelating groups.

### 3.2. Effect of the Introduced bPEI onto Cu^2+^ Removal Efficiency

The adsorption properties of the introduced nanoadsorbent were tested by taking different aspects into account, i.e., (i) The effect of solution pH and (ii) The initial concentration of Cu^2+^. In both cases, covalently linked amino-rich polymer nanocomposite was compared to MNPs@SiO_2_ in order to elucidate the effect of the introduced functional groups. Our preliminary studies (estimating the industry utilization), showed that the the optimal adsorbent (i.e., silica-coated magnetic NPs functionalized with carboxymethylchitosan) dosage was 0.1 mg·mL^−1^ and the removal time 1 hour. Therefore, these two parameters were assumed to be thoroughly optimal and were not examined.

#### 3.2.1. Effect of pH

**Figure 8:**
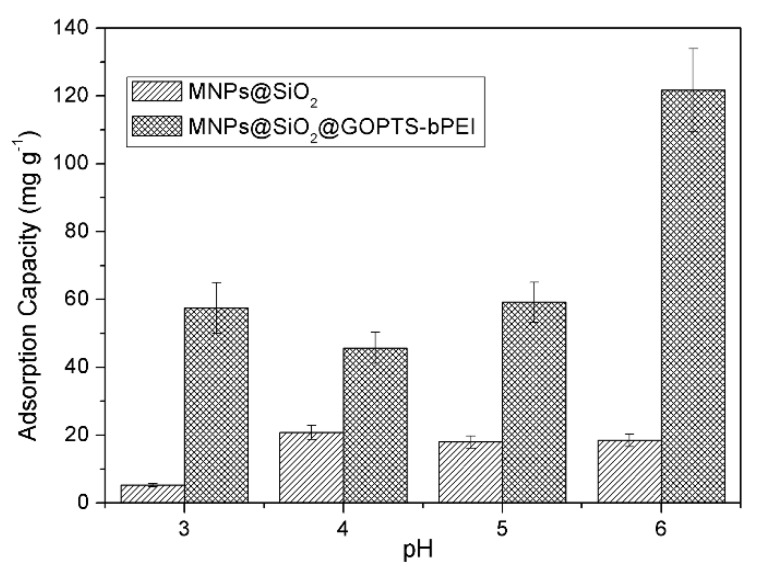
Influence of solution pH on adsorption capacity of Cu^2+^ for MNPs@SiO_2_ and GOPTS-bPEI modified MNPs@SiO_2_.

When taking into consideration the effect of pH, protonation and surface chemistry of the nanoadsorbent, together with the metal ion species distribution, plays an important role (Figure 8). It is known that the Cu^2+^ starts to form copper hydroxide precipitates at pH > of 6 [49]; therefore, the influence of the pH on the adsorption capacity was studied at pH = 6. Electrokinetic measurements (Figure 5a) showed that the IEP is located at around pH 8.5. Regarding this and the determined pK values for primary groups of bPEI (Figure 5b), it can be supposed that, at pH = 6, most of the primary amines are in deprotonated form. Therefore, they are highly accessible, due to the electron-donor pair with favorable Cu^2+^ chelation [50]. Therefore, larger Cu^2+^ adsorption capacity can be seen at pH = 6 when taking into account that the copper uptake mechanism most likely occurs through deprotonated primary groups [51], indicating the higher sorption of the Cu^2+^. At lower pH (pH < 6), the adsorption capacity is almost constant (~60 mg·g^−1^). This also correlates well with the pH-dependent titration and ZP behavior (Figure 4a,b), where most of the amino functional groups are in protonated state. Therefore, due to the presence of –NH_3_^+^ groups, the competition of H^+^ with the Cu^2+^ for binding sites can also be present. As already reported, the latter is more pronounced at lower pH values [17]. Beside this, the electrostatic repulsions between both positively charged MNPs@SiO_2_@GOPTS-bPEI and Cu^2+^ can, in part, hinder the Cu^2+^ adsorption. Different behavior can be observed for MNPs@SiO_2_. At pH = 3, the adsorption capacity of MNPs@SiO_2_ is approximately 5 mg·g^−1^. Adsorption efficiency increased with rising pH, most likely due to the electrostatic interactions between the negatively charged silanol groups and Cu^2+^. Nevertheless, the adsorption capacity of the GOPTS-bPEI-based adsorbent was 6-times higher at pH = 6 with respect to MNPs@SiO_2_ (Figure 8), indicating the advantage of covalently introduced derived-bPEI polymer. 

#### 3.2.2. Effect of Initial Cu^2+^ Concentration

**Figure 9:**
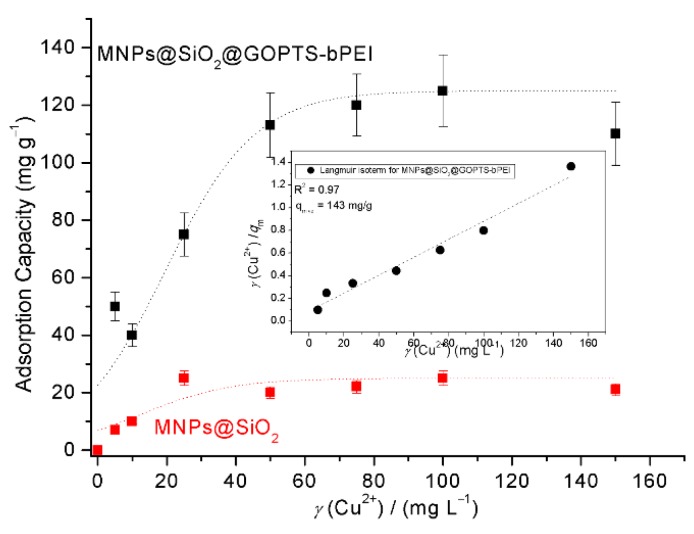
Effect of the initial Cu^2+^ concentration on the adsorption capacity for unmodified and GOPTS-bPEI modified MNPs@SiO_2_.

The influence was evaluated of initial Cu^2+^ concentration on adsorption efficiency (Figure 9). Adsorption capacity increased with upturning of the Cu^2+^ solution to the plateau part, where the Cu^2+^ in the solution is in balance to the Cu^2+^ onto the adsorbent, occupying all the amino groups. This may be the result of the difference in the concentration gradient between Cu^2+^ in the initial solution and its absence on the nanoadsorbent, acting as a driving force until all the active sorption places are overtaken. The obtained results were well fitted with the Langmuir isotherm (inset in Figure 9), suggesting the monolayer coverage of Cu^2+^ onto the nanoadsorbent during the adsorption process. The maximum adsorption capacity was shown to be 143 mg·g^−1^ and is one of the largest in comparison to the few previously studied PEI-based adsorbents for Cu^2+^ removal applications [44,51,52,53,54,55]. The latter can be attributed to the high density, and to the homogeneous distribution of amino groups from bPEI, as well as to the high specific surface area and porous structure. The role of surface modification was additionally compared to MNPs@SiO_2_ adsorption tendencies (Figure 9) and clearly elucidates the significant sorption capabilities with covalently introduced GOPTS-bPEI for HM removal.

#### 3.2.3. Reusability Cycles for Regeneration Studies

**Figure 10:**
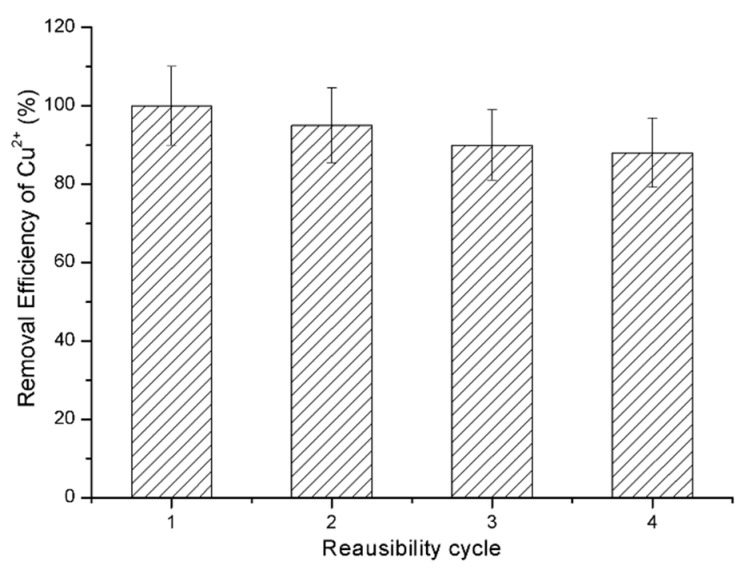
Reusability cycles of MNPs@SiO_2_@GOPTS-bPEI, expressed as adsorption–desorption studies, for copper removal.

Regeneration is an important indicator of nanoadsorbent economical attractiveness that contributes additionally to the waste minimization concept, where the same adsorbent can be used several times. It is determined mainly via adsorption–desorption experiments [24], and was calculated with Equation 2. In order to achieve effective desorption of HMs, and to apply adsorbents further to the next adsorption process, eluents with high desorption ability are exceptionally desirable. In our case, Na_2_EDTA was considered as the most appropriate eluent for regeneration studies, whilst it has already shown high desorption capability of the Na_2_EDTA usage as an eluent in the case of Cu^2+^ [38]. Figure 10 shows that the removal efficiency of the amino-rich nanoadsorbent decreased only to 95% after the second reusability cycle. MNPs@SiO_2_@GOPTS-bPEI still showed high adsorption tendencies for Cu^2+^ (88% of removal efficiency, Figure 10), even after the fourth adsorption–desorption cycle. This indicates possible multiple regeneration cycles of the nanoadsorbent, together with superior removal efficiency.

### 3.3. Proposed Possible Adsorption Mechanism for Cu

**Figure 11:**
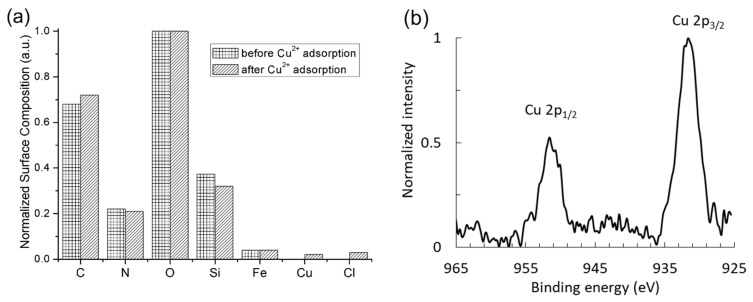
Normalized surface composition before and after Cu adsorption of MNPs@SiO_2_@GOPTS-bPEI (normalized to oxygen) is shown in (**a**) while the high-resolution core spectra for Cu 2p is shown in (**b**).

The adsorption mechanisms of Cu onto nanoadsorbent were explained using different surface analytical techniques. XPS surface technique revealed the presence of Cu after adsorption at the MNPs@SiO_2_@GOPTS-bPEI surface (Figure 11), underscoring its uptake after sorption. The surface composition before and after Cu uptake was normalized relative to oxygen (Figure 11a). It can be observed that the surface composition did not change significantly after Cu adsorption, only the presence of two additional elements was detected (i.e., Cu and Cl). Cu originates as a consequence of adsorption, while Cl originates from the copper model solution, and is most likely attached to MNPs@SiO_2_@GOPTS-bPEI via electrostatic attraction, despite magnetic separation and washing. Similar surface composition before and after Cu uptake shows clearly nanoadsorbent stability, and no desorption of the introduced derived amino-polymer. The high-resolution core spectrum for Cu 2p is shown in Figure 11b. The copper peak Cu 2p_3/2_ is located at the binding energy 933 eV, and can be correlated to Cu^0^ along with Cu^+^, as both are present at the similar bonding energy, and it is difficult to distinguish between them. However, the presence of Cu^2+^ after uptake can be excluded, as the typical peak for Cu^2+^ should be present at 933.5–934 eV. Moreover, typical satellite peaks in the area 940–945 eV should be observed [42]. None of these were seen in our case. The reduced electronic state of Cu can be attributed to the filling of the empty Cu atomic orbital with free electron pairs from the deprotonated amino groups [51]. This is in agreement with the Langmuir sorption model fits (inset in Figure 9), indicating monolayer adsorption, and suggesting that the adsorption mechanism corresponds to the chemisorption, together with the prediction that the adsorption occurred mainly due to the complexation of the Cu ions with the bPEI amine groups. Additionally, the authors in Reference [56] proposed calculations for different models that predict the manner of the metal ion bonding to the amine active adsorption sites. Taking their calculation with our parameters into consideration revealed the “pendant model”, which indicates that the chelation of copper with only one amino group is more suitable to describe the mechanism of Cu adsorption onto MNPs@SiO_2_@GOPTS-bPEI in the present study.

**Figure 12:**
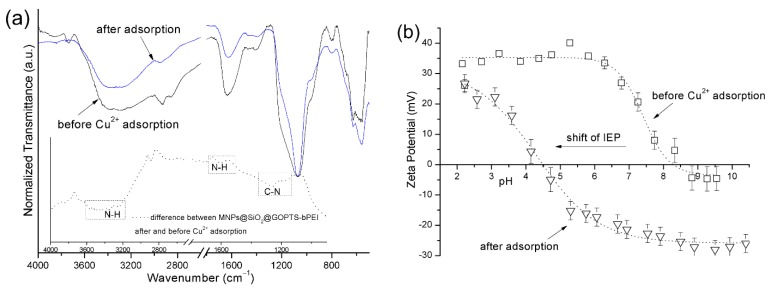
Attenuated Total Reflection (ATR) FTIR spectra before and after Cu adsorption with corresponding difference between them (**a**), and Zeta Potential behavior as a pH function for MNPs@SiO_2_@GOPTS-bPEI before and after Cu adsorption (**b**).

The MNPs@SiO_2_@GOPTS-bPEI were used as a Cu adsorbent at optimal conditions, and Cu uptake was characterized in terms of ATR-FTIR (Figure 12a). It was clearly observed that the major changes after Cu adsorption varied at the wavenumbers, typical for –NH vibrations. This was confirmed additionally with subtracting the FTIR spectra before and after Cu adsorption (inset in Figure 12a). With respect to that fact, it can be assumed that these functional groups are involved during Cu adsorption (complexation mechanism), and cause changes in vibrations of these functional groups. Similar observations were reported in Reference [17], and also support the observation from the XPS analysis above.

The electrokinetic measurements of the ZP were directly correlated to the surface potential behavior after Cu uptake by the amino-nanoadsorbent (Figure 12b). ZP measurements in a wide pH range showed the shift of the IEP after Cu adsorption to lower pH values (pH 4.5) in comparison to the pure adsorbent (pH 8.5). It can be assumed that the Cu is sharing free electron pairs with the amino groups from MNPs@SiO_2_@GOPTS-bPEI, and the latter are not accessible anymore for the protonation to express a highly positive charge. Moreover, when Cu is adsorbed onto MNPs@SiO_2_@GOPTS-bPEI, its more acidic character may be connected to the presence of Cu acting as an acid [57].

## 4. Conclusions

This paper represents for the first time the systematic concept of crosslinking epoxy-containing organosilane with branched PEI through a ring-opening reaction, and its further silanization onto silica-coated MNPs for copper removal applications through possible chelation mechanisms via deprotonated amino groups. The synthesized nanoadsorbent was composed of 13 nm-sized magnetic cores, uniformly covered with an approximately 3-nm-thick amorphous silica layer, and additionally silanized with GOPTS-derived bPEI. FTIR analysis revealed the presence of typical functional groups after silanization, as well as the XPS and TGA showed high content of N, suggesting an -NH_2_ rich adsorbent surface. Moreover, correlations between electrokinetic measurements and pH-potentiometric titrations were in high agreement with the proposed bonding mechanism. Additionally, the large magnetic response and precisely managed thickness of the surface layers showed quick removal after the finished adsorption process, which is highly appreciated for a successful adsorption process. In order to evaluate the efficiency of the introduced adsorbent, the effect of initial pH and the initial Cu^2+^ solution concentration were studied, and compared with respect to MNPs@SiO_2_. Results revealed the importance of surface modification, pH-dependency of the copper adsorption, and the monolayer coverage with large adsorption capacity. Moreover, after reusing the adsorbent, the latter shows high tendency towards Cu removal, even after several cycles. The proposed adsorption mechanism revealed major involvement of the NH/NH_2_ functional groups by the adsorption of copper by reducing its electronic state by donating free electron pairs. 

To conclude, the developed amino-rich nanoadsorbent represents an efficient, environmentally friendly, stable and straightforwardly prepared adsorbent for metal removal, especially in more complicated environmental conditions, such as sludge.

## Figures and Tables

**Table 1:** Atomic concentrations determined with XPS for MNPs, core-shell MNPs@SiO_2_, and GOPTS-bPEI modified MNPs@SiO_2_.

	C	N	O	Si	Fe
MNPs	15.4		53.2		31.4
MNPs@SiO_2_	7.4		65.4	24.4	3.3
MNPs@SiO_2_@GOPTS-bPEI	29.5	9.4	43.4	16.0	1.9

**Table 2:** Brunauer–Elmett–Teller (BET) surface area and pore volume for differently modified MNPs with respect to bare MNPs.

	BET Surface Area * (m^2^·g^−1^)	BJH Adsorption Pore Volume (cm^3^·g^−1^)
MNPs	13.56	/
MNPs@SiO_2_	25.21	0.16
MNPs@SiO_2_@GOPTS-bPEI	38.33	0.23

* the BET surface areas are smaller compared to the calculated theoretical specific surface area due to the high degree of agglomeration during the drying process.
